# Decoding neutrophil extracellular traps and key gene drivers in unexplained pregnancy loss

**DOI:** 10.3389/fimmu.2025.1628337

**Published:** 2025-09-08

**Authors:** Hui Ding, Lu Zhang, Wei Yang, Yu Liu, Chao Wang, Li Liu, Cheng Li, Liyuan Pan, Lin Chen, Meimei Liu

**Affiliations:** ^1^ Department of Obstetrics and Gynecology, The Second Affiliated Hospital of Harbin Medical University, Harbin, China; ^2^ Department of Reproductive Medicine Center, Zhoukou Central Hospital, Zhoukou, China; ^3^ Department of Obstetrics and Gynecology, Harbin Red Cross Central Hospital, Harbin, China

**Keywords:** neutrophil extracellular traps (NETs), unexplained recurrent pregnancy loss (uRPL), immune microenvironment, machine learning algorithms, decidual inflammation

## Abstract

**Background:**

Recurrent pregnancy loss (RPL) represents a critical reproductive health concern, with nearly half of RPL cases lacking clinically identifiable etiologies, termed unexplained RPL (uRPL). Neutrophil extracellular traps (NETs), released by activated neutrophils, have been implicated in the pathogenesis and progression of various reproductive disorders. However, the relationship between NETs and uRPL remains poorly characterized.

**Methods:**

This study enrolled 34 patients with uRPL and 30 healthy controls. Serum NETs biomarkers (MPO-DNA, citH3) were quantified via ELISA. Decidual tissues underwent histopathology (H&E), immunohistochemistry, and transcriptomics (6uRPL vs. 5 controls). Machine learning identified key NETs-related differentially expressed genes, validated by Western blotting. Immune cell infiltration and gene-immune correlations were assessed bioinformatically.

**Results:**

uRPL patients exhibited elevated serum NETs biomarkers (MPO-DNA, citH3; p<0.01) and increased decidual neutrophil infiltration. Immunohistochemistry confirmed upregulated MPO and citH3 in uRPL (p<0.01). Transcriptomics identified four key DE-NRGs (C3AR1, ITGAM, ITGB2, LYZ), validated at the protein level (p<0.05). Immune profiling revealed increased CD8+ T cells, M2 macrophages, and neutrophils, alongside reduced CD4+ memory T cells, follicular helper T cells, and monocytes in uRPL. All DE-NRGs correlated positively with M2 macrophages (r>0.6) and negatively with follicular helper T cells and monocytes (r<-0.5). LYZ also correlated with neutrophils (r>0.5). A nomogram incorporating DE-NRGs demonstrated robust diagnostic accuracy (AUC>0.85).

**Conclusion:**

This study establishes a novel link between NETs and the pathogenesis of uRPL. It highlights the abnormal activation of C3AR1, ITGAM, ITGB2, and LYZ, along with M2 macrophage polarization, as crucial factors in decidual immune dysregulation. These findings suggest that NETs could serve as therapeutic targets, while DE-NRGs may act as potential biomarkers for uRPL.

## 1 Introduction

Recurrent pregnancy loss (RPL)is a distressing pregnancy disorder experienced by ~2.5% of women trying to conceive (1). Defined as the failure of two or more clinically recognized pregnancies before 20–24 weeks of gestation, and includes embryonic and fetal losses (1). The etiology of RPL is multifactorial, involving genetic anomalies, endocrine disorders, anatomical abnormalities, infectious diseases, thrombophilic disorders, and immune dysregulation. However, nearly 50% of cases remain unexplained even after extensive clinical evaluation (2). Unexplained RPL (uRPL) presents a major clinical challenge, as the lack of identifiable causes hampers the development of targeted treatments. Emerging evidence emphasizes the crucial role of dysregulated maternal-fetal immune interactions and abnormal inflammatory responses in pregnancy maintenance and loss (3). Among the immune mechanisms implicated, neutrophil extracellular traps (NETs)—web-like structures composed of DNA, histones, and antimicrobial proteins released by activated neutrophils—have recently emerged as key players in both physiological defense and pathological inflammation (4, 5). While NETs are essential for combating infections, their excessive or dysregulated formation, termed NETosis, has been linked to autoimmune diseases, thrombotic disorders, and obstetric complications such as preeclampsia and preterm birth (6, 7). However, the contribution of NETs to uRPL, particularly their interplay with decidual immune cell dynamics and molecular pathways, remains poorly characterized. This study addresses this gap by integrating multi-omics and immune profiling to elucidate the role of NETs in uRPL pathogenesis.

Neutrophils, the most abundant leukocytes in human blood, rapidly respond to inflammatory stimuli by releasing NETs, which immobilize pathogens but also exacerbate tissue damage and inflammation (4). In pregnancy, neutrophils infiltrate the decidua and participate in immune tolerance and placental development (8). However, dysregulated NETosis has been implicated in adverse pregnancy outcomes. For instance, elevated NET biomarkers, such as myeloperoxidase-DNA (MPO-DNA) complexes, Neutrophil Elastase (NE), and citrullinated histone H3 (citH3), are observed in preeclampsia and spontaneous preterm birth, correlating with placental inflammation and vascular dysfunction (6, 9, 10). NETs may impair trophoblast invasion, activate complement pathways, and promote thromboinflammatory cascades, all of which could disrupt pregnancy (11). Despite these advances, the role of NETs in uRPL—a condition characterized by recurrent, often idiopathic losses—remains underexplored.

RNA sequencing (RNA-seq) has emerged as a pivotal tool for investigating disease pathogenesis due to its high sensitivity in resolving complex transcriptional regulatory networks (12). Methodological advancements in RNA-seq have significantly enhanced the accuracy of biomarker discovery, while the translational value of transcriptome analysis in therapeutic development is increasingly recognized (13, 14), This technology enables the detection of subtle, biologically critical transcriptomic alterations underlying complex diseases (15, 16).

Here, we investigated the contribution of aberrant NETs formation to the pathogenesis of uRPL by disrupting maternal-fetal immune homeostasis. Utilizing a multidimensional strategy integrating histopathological, transcriptomic, bioinformatic, and clinical data, we delineated the role of NETs in uRPL.

## 2 Materials and methods

### 2.1 Ethical approval and participant selection

This study was approved by the Ethics Committee of The Second Affiliated Hospital of Harbin Medical University (No. YJSKY2024-380). All participants provided written informed consent. From June to October 2024, 34 uRPL patients (aged 20–40 years, gestational age 35–66 days) and 30 healthy controls (HC; aged 21–37 years, gestational age 37–62 days) were enrolled. RPL was defined as ≥2 consecutive pregnancy losses before 24 weeks (excluding ectopic/molar pregnancies) (17). Controls had prior term live births and normal current pregnancies. Exclusion criteria: uterine anomalies, parental chromosomal abnormalities, immune/thyroid dysfunction, or systemic comorbidities (diabetes, hypertension, etc.).

### 2.2 Sample collection

Serum samples were collected from residual clinical specimens in EP tubes and stored at -80 °C, alongside clinical data (age, body mass index [BMI], obstetric history). Decidual tissues were obtained during dilation and curettage (D&C) following ultrasound-confirmed fetal demise in uRPL patients or elective termination in healthy controls. Decidual tissues from uRPL patients were included in subsequent analyses only after exclusion of fetal chromosomal abnormalities. Each tissue sample was divided into three portions: one fixed in 4% paraformaldehyde (24–48 hours, 4 °C) for paraffin embedding, and two snap-frozen in liquid nitrogen for storage at -80 °C. Procedures adhered to strict aseptic protocols.

### 2.3 ELISA

Serum NETs levels were assessed using MPO-DNA and citH3 as biomarkers, quantified via ELISA kits (MPO-DNA ELISA kit, Cat# SBJ-H26239; citH3 ELISA kit, Cat# SBJ-HI333; Zhengzhou Yizeng Biotechnology Co., Ltd.). All procedures followed the manufacturer’s protocols.

### 2.4 H&E staining and immunohistochemistry

To assess neutrophil infiltration in uRPL decidua, paraffin-embedded tissues were subjected to H&E staining for histological analysis. Immunohistochemistry (IHC) was performed to detect MPO (primary antibody, Cat# GB11224) and cit H3 (CitH3, primary antibody, Cat# GB12102) using HRP-conjugated secondary antibodies (goat anti-rabbit IgG, Cat# GB23303; goat anti-mouse IgG, Cat# GB23301; Servicebio).

### 2.5 RNA-sequencing

Decidual tissues from 11 patients (6 uRPL and 5 healthy controls) underwent RNA-seq(Sangon Biotech, Shanghai, China). Baseline characteristics are detailed in **Supplementary Table 1**. Total RNA was extracted using Trizol (Cat# B511311), quantified with Qubit 2.0 RNA Assay Kit (Life Technologies, Cat# Q32855), and enriched for mRNA using oligo-dT. Libraries were prepared via cDNA synthesis, PCR amplification, and sequenced on the DNBSEQ-T7 platform (MGI). Data quality was assessed using FastQC (v0.11.2), filtered with Trimmomatic (v0.36), and aligned to the GRCh38 genome via HISAT2 (v2.0). Normalization and analysis were performed using DESeq2.

### 2.6 Data analysis

#### 2.6.1 Differential expression analysis

Differentially expressed genes (DEGs) were identified using the “limma” package with thresholds of |logFC| > 0.585 and adjusted *p*< 0.05 (18). Visualization was performed using R packages “heatmap” and “ggplot2.”

#### 2.6.2 Weighted gene co-expression network analysis

Weighted Gene Co-expression Network Analysis (WGCNA) was performed using the “WGCNA” R package to identify gene modules associated with uRPL. Outliers were removed using the “hclust” function, and the optimal soft threshold was determined via “pickSoftThreshold.” An adjacency matrix was transformed into a topological overlap matrix (TOM), and gene modules were identified. Genes most closely associated with uRPL were selected from relevant modules.

#### 2.6.3 Identification and enrichment analysis of DE-NRGs

NETs-related differentially expressed genes (DE-NRGs) were identified by intersecting RPL-associated genes from WGCNA with DEGs and NETs-related genes (NRGs)(n=198) (19–21), (**Supplementary Table 2**). Gene Ontology (GO) and Kyoto Encyclopedia of Genes and Genomes (KEGG) enrichment analyses were performed using R packages (“org. Hs.eg.db,” “clusterProfiler,” “enrichplot”) and visualized via “ggplot2,” “GOplot,” and “circlize.” Gene Set Variation Analysis (GSVA) was applied to evaluate pathway activity changes related to NETs in uRPL patients (22). Using R packages (“msigdbr,” “GSVA,” “GSEABase,” “BiocParallel,” “tinyarray,” “Hmisc”), biological pathways were assessed, and results were visualized via “pheatmap,” “ggplot2,” “ggthemes,” and “ggprism.”

#### 2.6.4 Identification of core DE-NRGs and PPI network construction

Core DE-NRGs were identified using three machine learning methods: Least Absolute Shrinkage and Selection Operator (LASSO) (“glmnet” package) (23), Support Vector Machine Recursive Feature Elimination (SVM-RFE) (“e1071,” “kernlab,” “caret” packages) (24), and Random Forest(RF) (“randomForest,” “Boruta” packages). Overlapping genes from these methods were selected as key DE-NRGs. Protein-protein interaction (PPI) networks were constructed using the STRING database(https://cn.string-db.org).

#### 2.6.5 Nomogram model construction based on core DE-NRGs

Receiver operating characteristic (ROC) curves were generated using the “pROC” package to validate core DE-NRGs (25). A nomogram model was constructed using the “survival” and “rms” R packages, with calibration and decision curves plotted via “PredictABEL” and “rmda” to assess model performance, providing a simplified predictive tool for clinicians.

#### 2.6.6 Immune cell infiltration analysis using CIBERSORT

CIBERSORT, a computational method for estimating cell composition in complex tissues (26), was applied to assess immune cell infiltration differences between uRPL and control groups based on gene expression data.

### 2.7 Western blot

To validate the expression of core DE-NRGs-related proteins, Western blot analysis was performed as previously described (27). Briefly, collected decidual tissues were homogenized and lysed in ice-cold RIPA lysis buffer (G2002, Servicebio). The lysates were centrifuged at 12,000 g for 10 min at 4 °C, and the supernatants were collected immediately. Protein concentrations were determined using a BCA assay kit (G2026-200T, Servicebio) according to the manufacturer’s instructions. Equal amounts of protein were separated by 10% SDS-PAGE (G2075, Servicebio) and transferred onto PVDF membranes (WGPVDF45, Servicebio). After transfer, the membranes were briefly rinsed with TBST (G2150, Servicebio) and blocked with 5% non-fat milk in TBST for 30 min at room temperature with gentle shaking. Subsequently, the membranes were incubated with primary antibodies overnight at 4 °C. After washing with TBST, the membranes were incubated with HRP-conjugated secondary antibodies for 30 min at room temperature. Following another round of TBST washing, the membranes were placed on the chemiluminescence imaging system platform. ECL substrate (G2020, Servicebio) was added and incubated for 1 min, after which chemiluminescence signals were captured using the imaging system. The raw images were saved. Densitometric analysis was performed using ImageJ software (ImageJ, USA), with GAPDH serving as the loading control for normalization.

The primary antibodies used include the following:anti-GAPDH (GB15004, 1:10000, Servicebio), anti-ITGB2 (bsm-51539M, 1:1000, bioss), anti-C3AR1 (bs-2955R, 1:5000, bioss), anti-ITGAM (GB15058, 1:1000, Servicebio), and anti-LYZ (GB11345, 1:1000, Servicebio). Secondary antibodies used were HRP-conjugated goat anti-rabbit IgG (GB23303, 1:3000, Servicebio) and HRP-conjugated goat anti-mouse IgG (GB23301, 1:3000, Servicebio).

### 2.8 Statistical analysis

In this study, all statistical analyses were performed using R software (version 4.3.1) and GraphPad Prism (version 9.0). Continuous variables were presented as mean ± standard deviation (SD) or median (interquartile range, IQR) based on their distribution, which was assessed using the Shapiro-Wilk test. Comparisons between two groups were conducted using the Student’s t-test for normally distributed data or the Mann-Whitney U test for non-normally distributed data. Categorical variables were expressed as frequencies (percentages) and compared using the chi-square test or Fisher’s exact test, as appropriate. All statistical tests were two-sided, and p < 0.05 was considered statistically significant.

## 3 Results

### 3.1 Comparison of baseline characteristics between uRPL and HC

The study included 34 uRPL patients and 30 HC. No significant differences were observed in age, BMI, and gestational age (*p* > 0.05), while live birth history and number of pregnancy losses differed significantly between groups (*p* < 0.05) (**Table 1**).

**Table 1:** Clinical data of human subjects.

Variable	HC (n=30)	uRPL (n=34)	*t/z* value	*p* value
Age (Mean ± SD, year)	30.30 ± 3.88	32.21 ± 2.24	-1.87	0.067
BMI (Median (IQR), kg/m^2^)	22.46 (3.28)	23.23 (3.74)	-1.12	0.265
Number of live births (Media (IQR))	1 (1)	1 (1)	-3.09	0.002**^#^**
Days of pregnancy (Mean ± SD, days)	41.30 ± 8.09	43.29 ± 9.79	-0.881	0.382
Number of miscarriages (Median (IQR))	0 (0)	2 (1)	-7.48	0.000**^#^**

Comparison of baseline characteristics between uRPL and HC. ^#^ *P*< 0.05.

### 3.2 Increased NETs release and neutrophil infiltration in uRPL patients

**Figure 1:**
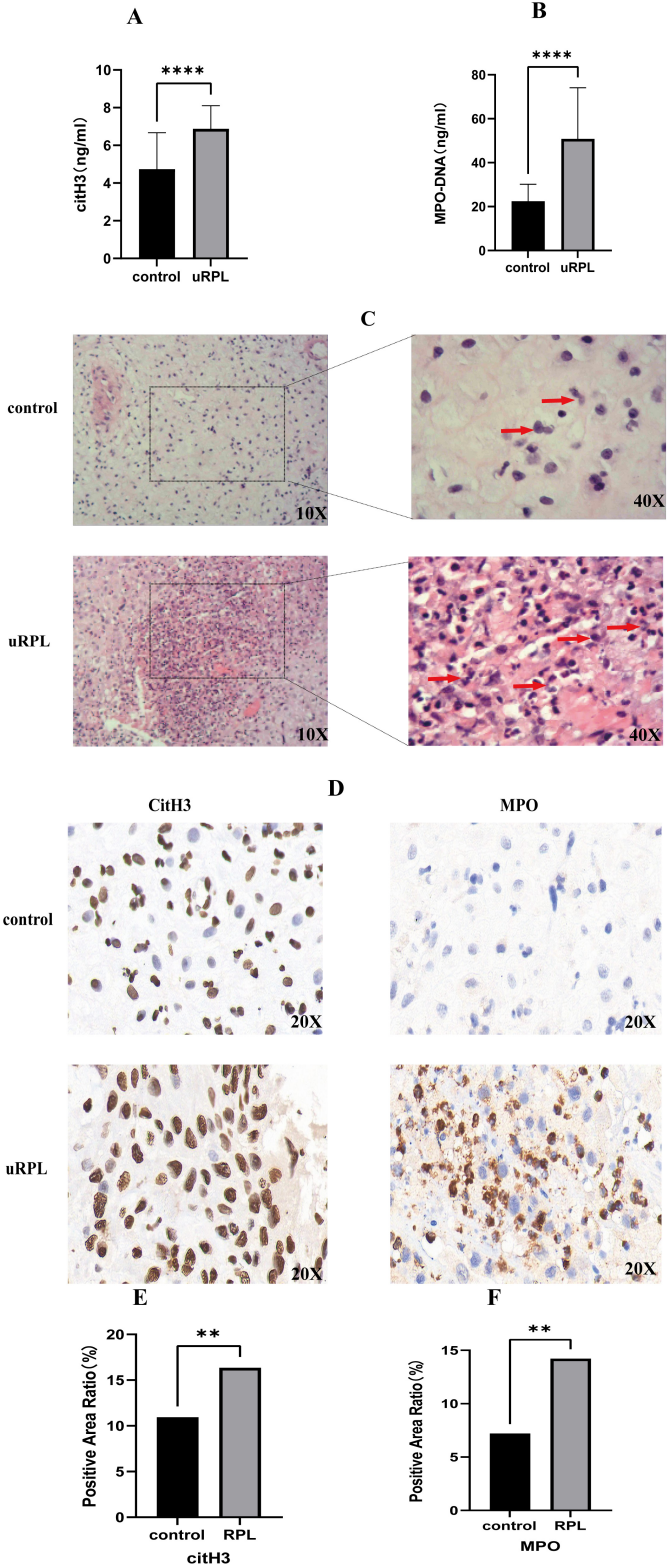
NETs formation and neutrophil infiltration in uRPL vs HC. **(A)** Serum citH3 levels. **(B)** Serum MPO-DNA levels. **(C)** H&E staining (neutrophils indicated). **(D-F)** IHC for citH3 **(D)**, MPO **(E)**, and quantitative intensity **(F)** **p < 0.01, **** p < 0.0001.

uRPL patients showed significantly higher serum levels of MPO-DNA (50.85 ± 20.28 ng/ml vs. 20.40 ± 7.78 ng/ml) and citH3 (4.74± 1.93 ng/ml vs.6.88± 1.22 ng/ml) compared to HC (*p* < 0.05; **Figures 1A, B**). H&E staining of decidual tissues from 3 uRPL and 3 HC cases revealed increased neutrophil infiltration in uRPL (**Figure 1C**). IHC confirmed elevated citH3 and MPO expression in uRPL decidua (*p*< 0.05; **Figures 1D-F**).

### 3.3 Sequencing and data analysis

To explore the correlation between NETs and uRPL and their potential mechanisms, we sequenced the decidual tissues of both groups to identify differentially expressed DE-NRGs. Baseline comparison between the two groups revealed that the uRPL group had significantly more fetal losses than HC, with no significant differences in age, BMI, gestational days, and live birth numbers. (**Supplementary Table 1**).

#### 3.3.1 Identification of key differentially expressed genes in uRPL

**Figure 2:**
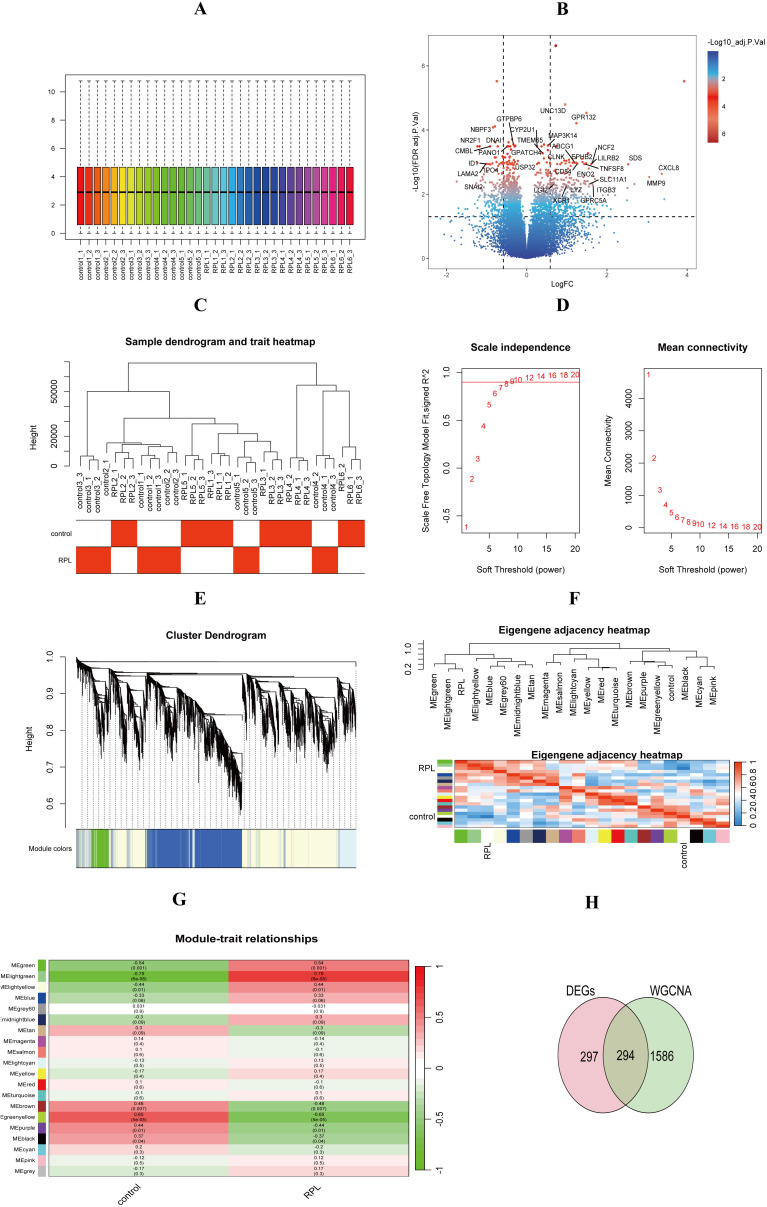
Identification of key differentially expressed genes in uRPL. **(A)** Normalized gene expression. **(B)** Volcano maps show differentially expressed genes. **(C)** Sample clustering dendrogram. **(D)** WGCNA soft-thresholding analysis. **(E)** Gene clustering dendrogram. **(F)** Gene module correlation. **(G)** Gene-clinical feature correlation. **(H)** Key gene intersection.

Sequencing data were normalized for subsequent analysis (**Figure 2A**). Using thresholds of adjusted *p*< 0.05 and |logFC| > 0.585, 591 DEGs were identified (402 upregulated, 189 downregulated) (**Figure 2B**). WGCN A analysis identified 20 co-expression modules, with 7 significantly associated with RPL (**Figures 2C-G**). We filtered genes using abs(geneModuleMembership) > 0.8 and abs(geneTraitSignificance) > 0.2, ultimately identifying 1,880 uRPL-related genes. Intersection with DEGs identified 294 key uRPL-associated genes (**Figure 2H**).

#### 3.3.2 Identification and enrichment analysis of DE-NRGs

**Figure 3:**
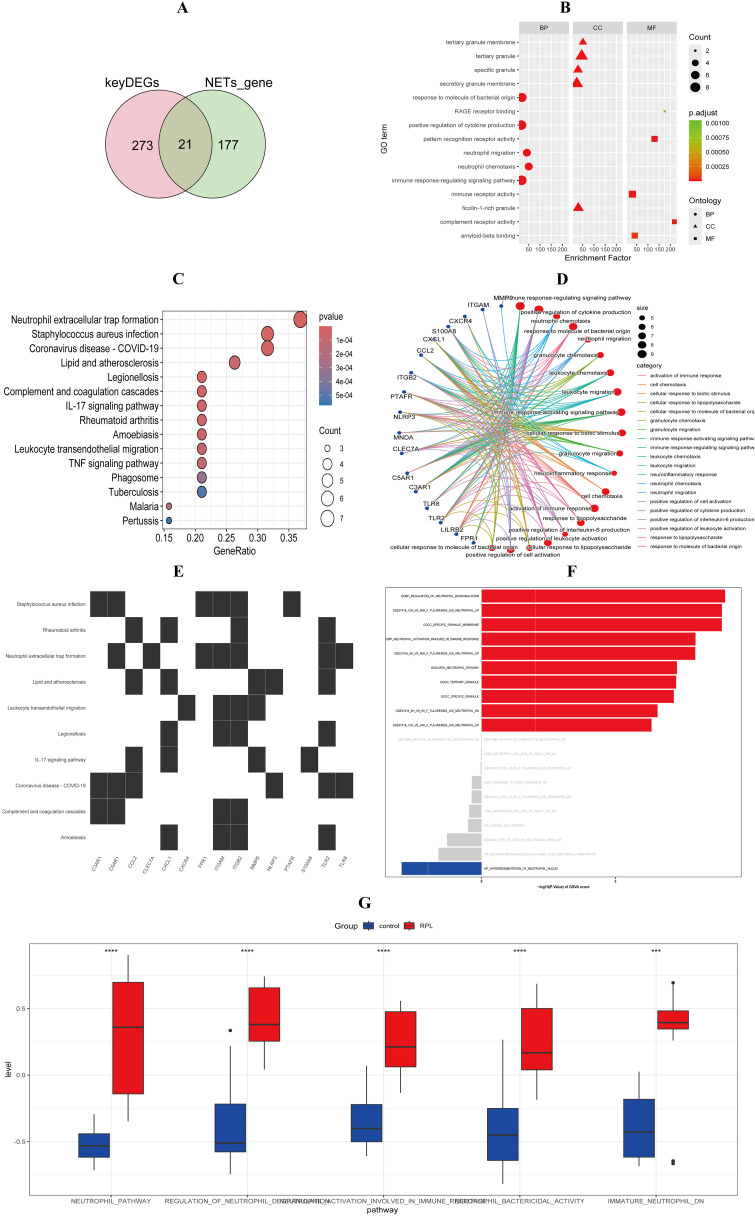
Identification and Enrichment Analysis of DE-NRGs. **(A)** 21DE-NRGs intersection. **(B)** GO enrichment. **(C)** KEGG enrichment. **(D)** GO term-gene network. **(E)** Gene-pathway heatmap. **(F, G)** NETs-related pathway activity comparison (HC vs uRPL). ***p < 0.001, ****p < 0.0001.

Intersection of 198 literature-derived NETs-related genes with 294 uRPL-associated genes identified 21 DE-NRGs (**Figure 3A**). GO enrichment analysis revealed significant enrichment in biological processes, including neutrophil chemotaxis, neutrophil migration, immune response-regulating signaling pathway, and positive regulation of cytokine production. Cellular components such as ficolin-1-rich granule, secretory granule membrane, specific granule, and tertiary granule membrane were also enriched. Molecular functions included amyloid-beta binding, complement receptor activity, immune receptor activity, pattern recognition receptor activity, and RAGE receptor binding (**Figure 3B**). KEGG analysis highlighted pathways such as neutrophil extracellular trap formation, Staphylococcus aureus infection, complement and coagulation cascades, IL-17 signaling pathway, leukocyte transendothelial migration, and TNF signaling pathway (**Figure 3C**). Relationships between DE-NRGs and GO terms or pathways are illustrated in **Figures 3D, E**, respectively. GSVA highlighted upregulated neutrophil activation and degranulation pathways in uRPL (**Figures 3F, G**).

### 3.4 Identification and validation of core DE-NRGs

**Figure 4:**
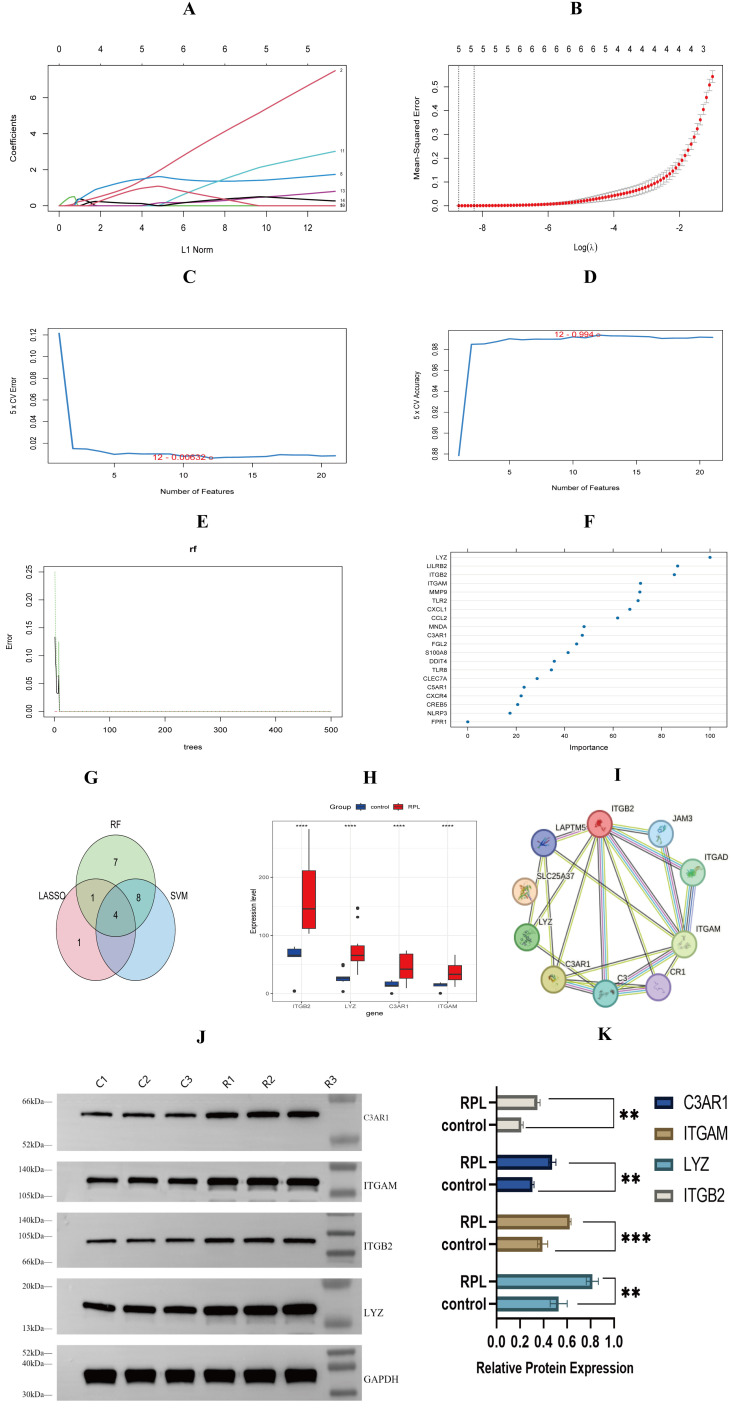
Machine learning identification and validation of core DE-NRGs. **(A, B)** LASSO regression identified 6 candidate DE-NRGs. **(C, D)** SVM-RFE identified 12 feature DE-NRGs. **(E)** Random forest error rate analysis. **(F)** Gene importance ranking. **(G)** Key DE-NRGs intersection (n=4). **(H)** Expression levels of ITGAM, ITGB2, LYZ, and C3AR1. **(I)** Key DE-NRG co-expression network. **(J, K)** Western blot validation of target proteins in decidual tissue (representative bands and quantification, n=3 per group). **p < 0.01, ***p < 0.001, ****p < 0.001.

To identify core DE-NRGs, we employed three machine learning algorithms: LASSO, SVM-RFE, and RF. LASSO analysis identified six candidate genes (**Figures 4A, B**), SVM-RFE selected twelve genes (**Figures 4C, D**), and RF highlighted twenty genes (**Figures 4E, F**). Intersection of these gene sets revealed four core DE-NRGs: ITGAM, ITGB2, LYZ, and C3AR1(**Figure 4G**). Comparative analysis demonstrated significant upregulation of these four DE-NRGs in the uRPL group compared to controls (**Figure 4H**). Protein co-expression analysis further supported their interaction (**Figure 4I**). Western blot validation in decidual tissues confirmed significantly higher expression levels of C3AR1, ITGAM, ITGB2, and LYZ in the uRPL group (**Figures 4J, K**). These findings highlight the potential role of these genes in uRPL pathogenesis. (summarized in **Table 2**).

**Table 2:** Core DE-NRGs in uRPL: biological functions and relevance to pathogenesis.

Gene Symbol	Full Name	Known Biological Functions	Expression in uRPL	Relevance to uRPL Pathogenesis	Potential Clinical Utility
C3AR1	Complement C3a Receptor 1	1. Mediates neutrophil chemotaxis and phagocytosis. 2. Activates complement signaling.	Upregulated (Protein level, WB)	1. Drives neutrophil infiltration and NETosis 2. Correlates with M2 macrophage polarization	Diagnostic biomarker (AUC = 0.867)
ITGAM	Integrin Alpha-M	1.Forms CD11b/CD18 (Mac-1) integrin. 2. Facilitates leukocyte adhesion and migration.	Upregulated (Protein level, WB)	1. Promotes pro-inflammatory decidual microenvironment. 2. Correlates with M2 macrophages and monocytes.	Diagnostic biomarker (AUC = 0.874)
ITGB2	Integrin Beta 2	1. Subunit of leukocyte integrins (e.g., Mac-1) 2. Critical for immune cell adhesion.	Upregulated (Protein level, WB)	1. Enhances immune cell adhesion in decidua. 2. Strongest diagnostic power.	Diagnostic biomarker (AUC = 1.000)
LYZ	Lysozyme	1. Antimicrobial enzyme 2. Innate immune defense	Upregulated (Protein level, WB)	1. Induces decidual inflammation 2. Correlates with neutrophils and M2 macrophages	Diagnostic biomarker (AUC = 0.959)

Biological functions of the key genes *C3AR1*, *ITGB2*, *ITGAM*, and *LYZ*, and their association with uRPL.

### 3.5 Diagnostic performance of core DE-NRGs and nomogram development

**Figure 5:**
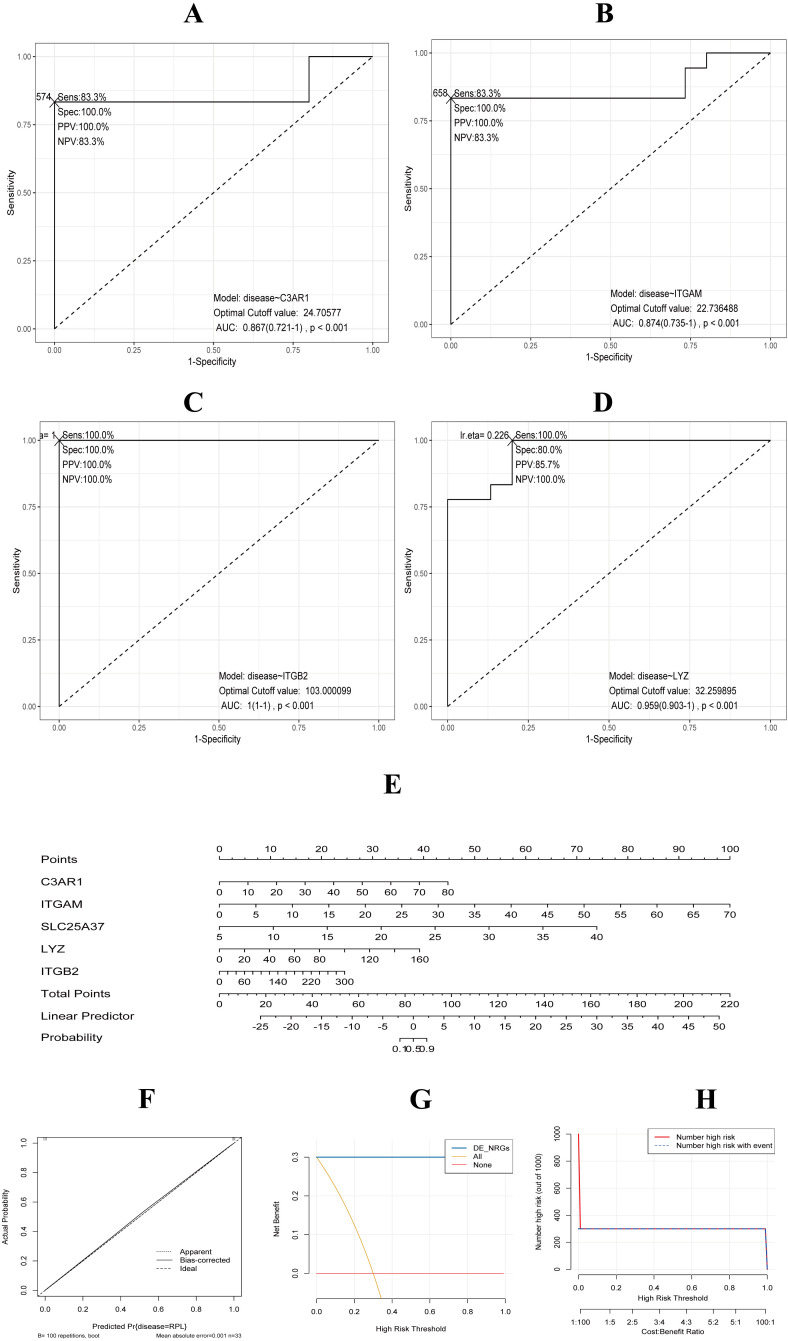
Development and validation of a prognostic nomogram based on core DE-NRGs. **(A-D)** ROC curves evaluating C3AR1, ITGAM, ITGB2, and LYZ. **(E)** Nomogram for predicting prognosis in uRPL patients. **(F)** Calibration curve showing agreement between nomogram predictions and actual outcomes. **(G)** Decision curve analysis assessing clinical utility. **(H)** Clinical impact curve.

We evaluated the diagnostic efficacy of the core DE-NRGs using ROC curve analysis. The ROC curves demonstrated excellent diagnostic performance for the gene biomarkers C3AR1, ITGAM, ITGB2, and LYZ. Specifically, C3AR1 showed an AUC of 0.867 (95% CI: 0.721–1.000) with a cut-off value of 24.71, sensitivity of 83.3%, and specificity of 100%; ITGAM demonstrated an AUC of 0.874 (95% CI: 0.735–1.000), a cut-off of 22.74, sensitivity of 83.3%, and specificity of 100%; ITGB2 achieved a perfect AUC of 1.000 (95% CI: 1.000–1.000) with a cut-off of 103.00, sensitivity of 100%, and specificity of 100%; and LYZ exhibited an AUC of 0.959 (95% CI: 0.903–1.000), a cut-off of 32.26, sensitivity of 100%, and specificity of 80.0%. (**Figures 5A–D**, **Supplementary Table S3**). Subsequently, we developed a NETs-related nomogram model based on 4 core DE-NRGs to provide clinicians with a streamlined and reliable diagnostic tool (**Figure 5E**). Calibration curve analysis demonstrated that the nomogram’s accuracy closely matched the actual positive rates (**Figure 5F**). Furthermore, decision curve analysis and clinical impact analysis indicated that our nomogram model could significantly assist in identifying uRPL, as illustrated in **Figures 5G, H**.

### 3.6 Differences in the immune characteristics of uRPL patients

**Figure 6:**
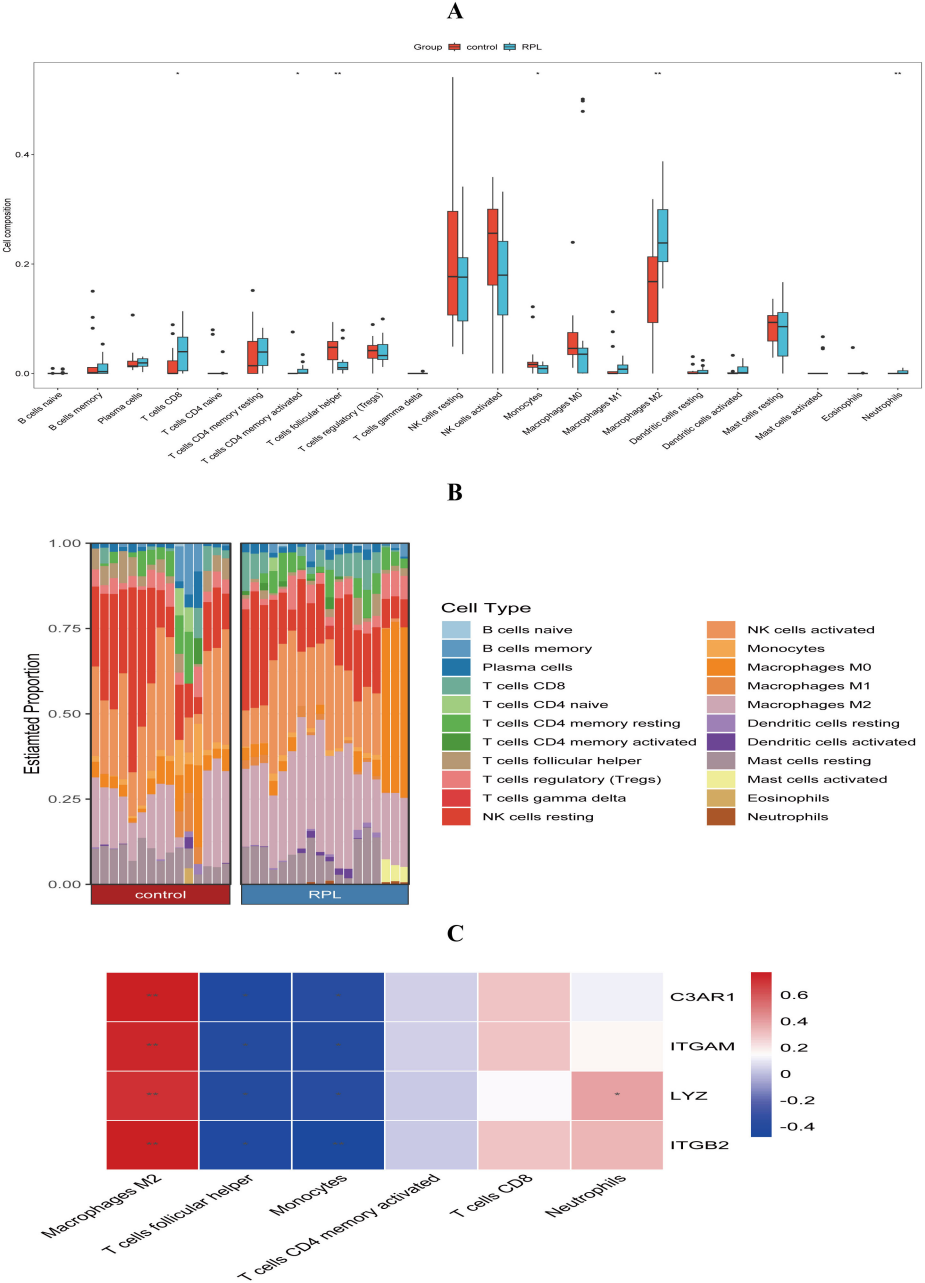
Comparison of immune infiltration between the uRPL and HC groups. **(A)** Infiltration levels of 22 immune cell types. **(B)** Proportions of immune cells. **(C)** Correlation between core DE-NRGs and immune cell infiltration changes. *p < 0.05, **p < 0.01.

We conducted immune infiltration analysis using the CIBERSORT algorithm. The results revealed increased infiltration levels of CD8 T cells, M2 macrophages, and neutrophils in the uRPL group, while activated memory CD4 T cells, follicular helper T cells, and monocytes were reduced in the uRPL group (**Figure 6A**). **Figure 6B** illustrates the infiltration levels of various immune cells across samples. Additionally, correlation analysis showed that all four core DE-NRGs were significantly positively correlated with M2 macrophages, negatively correlated with follicular helper T cells and monocytes, and LYZ was positively correlated with neutrophils (**Figure 6C**).

## 4 Discussion

uRPL poses significant clinical and psychological challenges, underscoring the urgent need to elucidate its underlying mechanisms. This study integrated clinical, histopathological, transcriptomic, and bioinformatic approaches to systematically explore the association between NETs and uRPL and their potential mechanisms. We first observed elevated serum NETs markers in uRPL patients, indicating aberrant neutrophil activation and NETs release, consistent with prior research linking NETs to pregnancy complication (10, 28). Histological analyses revealed increased decidual neutrophil infiltration, supported by immunohistochemistry, implicating neutrophil hyperactivation and NETs formation in decidual microenvironment imbalance. Previous studies have shown that NETs play a significant role in autoimmune diseases and thrombosis (29), and excessive release of NETs may disrupt maternal-fetal immune tolerance, leading to embryo loss (5). NETs-derived cfDNA exacerbates inflammation by inducing tumor necrosis factor-α (TNF-α) mRNA, while histones trigger apoptosis and act as damage-associated molecular patterns (DAMPs), promoting proinflammatory cytokine release, cytotoxicity, and ROS-mediated endothelial dysfunction, impairing embryo implantation and placental development, ultimately contributing to adverse pregnancy outcomes (7, 30).

Subsequent transcriptomic analysis of decidual tissues revealed significant upregulation of DE-NRGs. GO, KEGG, and GSVA enrichment analyses demonstrated that DE-NRGs were primarily involved in neutrophil chemotaxis, migration, activation pathways, degranulation regulation, Neutrophil extracellular trap formation, complement and coagulation cascades, and TNF signaling pathways, collectively contributing to RPL pathogenesis (**Figure 3**). Machine learning algorithms identified four hub DE-NRGs: C3AR1, ITGAM, ITGB2, and LYZ (**Figure 4**). These genes encode proteins involved in complement activation, leukocyte adhesion, and microbial defense (31–33), suggesting their potential roles in amplifying NETs-driven inflammation and immune dysregulation. Western blot confirmed elevated protein expression of hub DE-NRGs in uRPL decidual tissues, solidifying their association with disease pathology (**Table 2**).

C3AR1, a complement receptor central to the complement system, regulates C3a signaling to promote neutrophil chemotaxis and phagocytosis, influencing NETs formation (34, 35). Recent research links reduced placental C3AR1 expression in preeclampsia to maternal blood pressure, implicating the janus kinase–signal transducer and activator of transcription (JAK-STAT), transforming growth factor-β (TGF-β), and hypoxia-inducible factor-1 (HIF-1) pathways, as well as NK cell/M1 macrophage activity (36). However, our findings reveal upregulated C3AR1 in uRPL decidua, warranting further mechanistic exploration.

ITGAM and ITGB2 encode integrin αM (CD11b) and β2 (CD18) subunits, forming the CD11b/CD18 heterodimer (also known as aMb2, Mac-1, and CR3), which mediates myeloid cell adhesion, migration, and phagocytosis by binding ligands like ICAM-1 and fibrinogen, contributing to inflammation, immune defense, and thrombosis (37, 38). Dysregulation may promote proinflammatory immune responses, disrupting early pregnancy immune homeostasis and triggering adverse outcomes (39). Turunen et al. reported elevated CD11b in neutrophils/monocytes of infants born to early-onset preeclampsia, correlating with postpartum systemic inflammation (40). Yun-Long Zhang et al. demonstrated CD11b/CD18-mediated monocyte adhesion and macrophage polarization in cardiac remodeling (41). Our study further revealed positive correlations of ITGAM and ITGB2 with Macrophages M2 and negative correlations with monocytes in decidual tissues, but their mechanistic impact on endometrial decidualization remains unclear.

LYZ, a key antimicrobial enzyme of the innate immune system, plays vital roles in embryonic and neonatal immune defense (33). Natalia et al. reported upregulated serum LYZ levels in early-onset preeclampsia (42). In this study, marked LYZ elevation was observed in RPL decidual tissues, positively correlated with neutrophil infiltration. While no direct evidence links LYZ to pregnancy loss, these findings suggest LYZ-mediated decidual inflammation may contribute to uRPL pathogenesis.

Notably, ROC curves of the four hub DE-NRGs exhibited AUC values >0.85 (**Figures 5A-D**), indicating strong diagnostic potential for uRPL. Utilizing these markers, we constructed a nomogram model (**Figure 5E**), with the calibration curve demonstrating excellent agreement between predicted and observed probabilities (mean absolute error = 0.003). Decision curve and clinical impact curve analyses further validated its clinical utility (**Figures 5G, H**). Future efforts will initially expand the cohort at our center (n=100 uRPL patients), subsequently validate the model in multi-center cohorts, and ultimately integrate it with established RPL diagnostic indicators (e.g., maternal age) to construct a composite scoring system. This will be followed by developing a cloud-based platform for automated NETs-related risk assessment.

A key advancement of this study lies in integrating immune cell infiltration analysis with NETs-related molecular networks. Using CIBERSORT, we identified altered infiltration patterns of six immune subsets in uRPL: increased CD8+ T cells, M2 macrophages, and neutrophils, alongside decreased CD4+ activated memory T cells, follicular helper T cells, and monocytes. These findings contrast with prior reports. For instance, Changqiang Wei et al. observed reduced M1/M2 macrophage infiltration in RPL via GEO data analysis (43), while Yujia Luo et al. reported elevated monocytes and reduced T cells in RPL (44). Hui Hu et al. identified differences in eosinophils, monocytes, NK cells, and Tregs, likely reflecting cohort heterogeneity (45). The results of this study show that neutrophil infiltration in the decidual tissue of uRPL patients is increased, which is consistent with the HE staining results of the decidual tissue, and further validates the abnormal NETs formation in uRPL.

Macrophages, as key regulators of both innate and adaptive immune responses, can polarize into pro-inflammatory M1 macrophages or anti-inflammatory M2 macrophages under different microenvironmental conditions (46). M1 macrophages can be activated by lipopolysaccharide (LPS) and interferon-γ (IFN-γ), secreting inflammatory factors like TNF-α, interleukin (IL)-1β, and IL-6, to kill invading pathogens, perform phagocytosis, and clear aged or damaged cells (47, 48). M2 macrophages, activated by IL-4 and IL-13, secrete anti-inflammatory cytokines such as IL-10, IL-4, and transforming growth factor-β (TGF-β), inhibit T cell proliferation and activation, and participate in Th2 immune responses, aiding tissue repair and angiogenesis (48, 49). Therefore, the M1/M2 balance is crucial for tissue homeostasis. Our findings revealed increased M2 macrophage infiltration in uRPL, suggesting unresolved inflammation potentially exacerbated by aberrant NETs formation.

Accumulating evidence indicates that NETs promote inflammatory responses by interacting with various cells in the immune system. Exposure of macrophages to NETs triggers the activation of the NLRP3 inflammasome, facilitating the release of IL-1β and IL-18 (50). NETs also directly induce the secretion of other pro-inflammatory cytokines, including IL-8, IL-6, and TNF-α (51, 52). Research by Wang Y et al. demonstrated that neutrophils can promote the production of inflammatory cytokines in macrophages by triggering p65 nuclear translocation via activation of the TGF-β1/Smad signaling pathway (53). Integrative multi-omics analysis by Zhao YF et al. revealed that macrophages can coordinate NETs generation through the CXCL3/CXCR2 axis, thereby contributing to the progression of related diseases (54). Furthermore, studies by Kuang L et al. showed that macrophages, upon apoptosis, can transfer mitochondria to neutrophils via microvesicles; this process induces mitochondrial dysfunction and triggers NETs formation through the mitochondrial reactive oxygen species (mtROS)/Gasdermin D (GSDMD) axis (55). Collectively, these findings suggest a close relationship and robust crosstalk between NETs and macrophages.

Additionally, four hub DE-NRGs are positively correlated with M2 macrophages and negatively correlated with T cells, follicular helper and monocytes, while LYZ shows a unique positive correlation with neutrophils. We hypothesize that NETs-related genes regulate immune cell interactions, leading to macrophage polarization to the M2 phenotype, suppressing adaptive immunity, and negatively impacting inflammatory responses and immune tolerance during pregnancy, thereby increasing the risk of pregnancy loss.

Finally, this study has limitations. The modest cohort size restricted subgroup stratification; larger cohorts are needed to validate NETs dynamics across uRPL subtypes. Second, the limited transcriptomic samples (6 uRPL vs. 5 controls) may introduce bias despite using stringent thresholds (|logFC|>0.585, adjusted p<0.05) and validating key genes via WB. Third, we focused solely on transcriptional dysregulation of four NETs-related genes without exploring genetic variations or underlying mechanisms. Future investigations will address these limitations through a phased approach: We will first expand clinical cohorts to 50 uRPL and 50 controls, quantifying decidual DE-NRG expression (qPCR/WB/IHC) while locating NETs-gene co-expression via immunofluorescence, and correlating serum DE-NRG/NETs markers (MPO-DNA/citH3) with miscarriage history and gestational age. Subsequently, an *in vitro* decidual stromal cell-neutrophil coculture system will assess NETosis, apoptosis, and decidualization markers post-siRNA knockdown, followed by phosphoproteomic pathway screening. *In vivo* studies will administer NETosis inhibitors to classic RPL mice (CBA/J♀×DBA/2♂) to evaluate NETs dynamics and pregnancy outcomes. Finally, we will integrate spatial transcriptomics with single-cell epigenomics to map NETs microenvironment regulation while actively establishing a multicenter uRPL-specific cohort for nomogram validation and integrated genomic/methylation analyses to elucidate NETs’ mechanistic role in uRPL pathogenesis and clinical management.

## 5 Conclusion

This study demonstrated elevated NETs in serum and decidua of uRPL patients. Transcriptomic sequencing identified four hub DE-NRGs (C3AR1, ITGAM, ITGB2, LYZ), validated as protein-level markers. Decidual immune dysregulation was associated with these genes, characterized by altered neutrophil and macrophage infiltration. These findings reveal novel molecular mechanisms of NETs in uRPL pathogenesis, proposing NETs components and associated genes as potential biomarkers for early uRPL screening.

## Data Availability

The datasets generated and analyzed during the current study are available in the NCBI SRA repository under the BioProject accession number PRJNA1313132. The permanent link to the data is: https://www.ncbi.nlm.nih.gov/sra/PRJNA1313132.
