# Supplementary material for: Decoding neutrophil extracellular traps and key gene drivers in unexplained pregnancy loss
Source: Front Immunol. 2025 Sep 8;16:1628337. doi: 10.3389/fimmu.2025.1628337 (PMC12457963; doi:10.3389/fimmu.2025.1628337)
Supplement: Supplementary file 1 [file Table1.docx]

**Table S1 :** **Comparison of Baseline Data between uRPL and HC**

|  | Control(n=5) | RPL(n=6) | *t/z* value | *p* value |
| --- | --- | --- | --- | --- |
| Age（Mean±SD, year） | 29.80±5.81 | 31.17±5.24 | 0.419 | 0.685 |
| BMI（Mean±SD, kg/m^2^） | 22.19±0.88 | 23.49±1.63 | 1.59 | 0.146 |
| Days of pregnancy（Mean±SD, days） | 52.60±7.77 | 56.67±3.61 | 1.151 | 0.279 |
| Number of live births（Median（IQR）） | 1（0.5） | 1.5（1.5） | -0.604 | 0.63 |
| Number of miscarriages（Median（IQR）） | 0（0） | 2（0.5） | -3.228 | 0.02**^＃^** |

**Table S1 :Comparison of baseline characteristics between uRPL and HC. #***P*< 0.05.
